# Prostaglandin E_2_ Inhibits Histamine-Evoked Ca^2+^ Release in Human Aortic Smooth Muscle Cells through Hyperactive cAMP Signaling Junctions and Protein Kinase A[Fn FN3]

**DOI:** 10.1124/mol.117.109249

**Published:** 2017-11

**Authors:** Emily J. A. Taylor, Evangelia Pantazaka, Kathryn L. Shelley, Colin W. Taylor

**Affiliations:** Department of Pharmacology, University of Cambridge, Cambridge, United Kingdom

## Abstract

In human aortic smooth muscle cells, prostaglandin E_2_ (PGE_2_) stimulates adenylyl cyclase (AC) and attenuates the increase in intracellular free Ca^2+^ concentration evoked by activation of histamine H_1_ receptors. The mechanisms are not resolved. We show that cAMP mediates inhibition of histamine-evoked Ca^2+^ signals by PGE_2_. Exchange proteins activated by cAMP were not required, but the effects were attenuated by inhibition of cAMP-dependent protein kinase (PKA). PGE_2_ had no effect on the Ca^2+^ signals evoked by protease-activated receptors, heterologously expressed muscarinic M3 receptors, or by direct activation of inositol 1,4,5-trisphosphate (IP_3_) receptors by photolysis of caged IP_3_. The rate of Ca^2+^ removal from the cytosol was unaffected by PGE_2_, but PGE_2_ attenuated histamine-evoked IP_3_ accumulation. Substantial inhibition of AC had no effect on the concentration-dependent inhibition of Ca^2+^ signals by PGE_2_ or butaprost (to activate EP_2_ receptors selectively), but it modestly attenuated responses to EP_4_ receptors, activation of which generated less cAMP than EP_2_ receptors. We conclude that inhibition of histamine-evoked Ca^2+^ signals by PGE_2_ occurs through “hyperactive signaling junctions,” wherein cAMP is locally delivered to PKA at supersaturating concentrations to cause uncoupling of H_1_ receptors from phospholipase C. This sequence allows digital signaling from PGE_2_ receptors, through cAMP and PKA, to histamine-evoked Ca^2+^ signals.

## Introduction

Ca^2+^ and cAMP are ubiquitous intracellular messengers that regulate most cellular behaviors. The versatility of these messengers depends on both the spatiotemporal organization of the changes in their concentration within cells (Cooper and Tabbasum, 2014) and on interactions between them [see references in Tovey et al. (2008)]. These interactions are important in many smooth muscles, where increases in intracellular free Ca^2+^ concentration ([Ca^2+^]_i_) stimulate contraction, but receptors that stimulate formation of cAMP usually cause relaxation. The clinical importance is clear from the widespread use of *β*-agonists to provide symptomatic relief from asthma (Morgan et al., 2014). In vascular smooth muscle (VSM), too, cAMP attenuates the contractile responses mediated by many receptors that evoke Ca^2+^ signals (Morgado et al., 2012). This inhibition is assumed to be mediated by cAMP-dependent protein kinase (PKA) (Murthy, 2006), but there are also PKA-independent effects of cAMP (Spicuzza et al., 2001). At least some of these effects may be through exchange proteins activated by cAMP (EPACs), probably EPAC 1, which is abundant in blood vessels particularly within endothelial cells (Roscioni et al., 2011).

Histamine and prostaglandin E_2_ (PGE_2_) are two important inflammatory mediators. Their effects on VSM, which include regulation of proliferation (Yau and Zahradka, 2003) and vascular tone (Toda, 1987; Jadhav et al., 2004), are mediated by direct interactions with VSM and indirectly through release of autocrine signals from other cells (Norel, 2007). Histamine, PGE_2_ and their receptors are also implicated in vascular pathology, including inflammation (Norel, 2007), atherosclerosis (Gómez-Hernández et al., 2006), and restenosis (Sasaguri et al., 2005).

We demonstrated previously that histamine, through H_1_ receptors, stimulates an increase in [Ca^2+^]_i_ in human aortic smooth muscle cells (ASMC). The initial response is mediated by Ca^2+^ release through inositol 1,4,5-trisphosphate receptors (IP_3_R) and it is followed by Ca^2+^ entry across the plasma membrane (Pantazaka et al., 2013). PGE_2_, acting largely through EP_2_ receptors, both stimulates the activity of adenylyl cyclase (AC) and substantially attenuates the Ca^2+^ signals evoked by histamine. Here, we show that inhibition of histamine-evoked Ca^2+^ signals by PGE_2_ is mediated by cAMP delivered within “hyperactive signaling junctions.” The response does not require EPACs, but it is attenuated by inhibition of PKA. The effect of PGE_2_ on histamine-evoked Ca^2+^ signals does not result from a decrease in IP_3_R sensitivity or from increased Ca^2+^ extrusion from the cytosol, nor does PGE_2_ affect the Ca^2+^ signals evoked by stimulation of either endogenous type 1 protease-activated receptor (PAR1) or heterologously expressed muscarinic M3 acetylcholine receptors. We suggest that PKA uncouples H_1_ histamine receptors from the guanine nucleotide-binding protein, G_q/11_, and activation of phospholipase C (PLC). Our results establish that digital signaling from PGE_2_ receptors, through cAMP and PKA, inhibits histamine-evoked Ca^2+^ signals.

## Materials and Methods

### 

#### Materials.

H89, NKH 477, 8-Br-cAMP, and 8-Br-cGMP were from R&D Systems (Abingdon, Oxford, UK). *S*p-cAMPS, 6-Bnz-cAMP, 8-pCPT-2′-O-Me-cAMP, *R*p-cAMPS, *R*p-8-CPT-cAMPS, ESI-09, and HJC0197 were from Biolog (Bremen, Germany). Ionomycin, SQ 22536, DDA and myristoylated-PKA inhibitor peptide (PKI) were from Merck-Millipore (Watford, UK). A membrane-permeant peptide inhibitor of A kinase-anchoring proteins (AKAPs) [stearated Ht31 AKAP inhibitor peptide (st-Ht31)] and its proline-modified inactive form (st-Ht31P) were from Promega (Southampton, UK). Thapsigargin was from Alomone Laboratories (Jerusalem, Israel). PAR1 peptide, histamine dihydrochloride, forskolin, IBMX, and PGE_2_ were from Sigma-Aldrich (Welwyn Garden City, UK). Butaprost (free acid) and L902,688 were from Cayman Chemicals (Ann Arbor, MI). Membrane-permeant caged IP_3_ (ci-IP_3_PM) was from SiChem (Bremen, Germany). [2,8-^3^H] adenine ci-IP_3_PM, d-2,3-O-isopropylidene-6-O-(2-nitro-4,5-dimethoxy)benzyl-myo-inositol 1,4,5-trisphosphate-hexakis(propionoxymethyl) ester was from Perkin Elmer (Seer Green, Bucks, UK). Fluo-8 was from Stratech Scientific Ltd (Newmarket, Suffolk, UK). Other reagents were from Sigma-Aldrich, sources specified previously (Pantazaka et al., 2013) or identified in this section.

#### Culture of Human Aortic Smooth Muscle Cells.

Human ASMC from the American Tissue Culture Collection (Manassas, VA) or Dr. Trevor Littlewood (Boyle et al., 2002) were cultured as described (Pantazaka et al., 2013). Ethical approval for the latter was obtained from Addenbrooke’s NHS Trust. Cells were derived from four Caucasian patients (males aged 23, 52, and 54, and a female aged 58), who died of causes unrelated to cardiovascular pathologies. Cells were used between passages two and six.

#### Measurements of [Ca^2+^]_i_.

Histamine-evoked changes in [Ca^2+^]_i_ were recorded from cell populations using confluent cultures of ASMC grown in 96-well plates and loaded with Fluo-4 or Fluo-8. Experiments were performed in HEPES-buffered saline (HBS) at 20°C. HBS had the following composition (mM): NaCl 135, KCl 5.9, MgCl_2_ 1.2, CaCl_2_ 1.5, glucose 11.5, and HEPES 11.6 (pH 7.3). Fluorescence was recorded using a FlexStation 3 fluorescence plate-reader (MDS Analytical Technologies, Wokingham, UK) and calibrated to [Ca^2+^]_i_ as described (Pantazaka et al., 2013).

For measurements of [Ca^2+^]_i_ in single cells, confluent cultures of ASMC grown on poly-l-lysine-coated coverslips (22-mm diameter) were loaded with Fura-2 in HBS containing Fura-2 AM (4 *μ*M), probenecid (2.5 mM), and pluronic F127 (0.02% v/v) for 1 hour at 20°C. Fluorescence (detected at >510 nm with alternating excitation at 340 and 380 nm) was recorded using an Olympus IX71 inverted fluorescence microscope and Luca (electron-multiplying charge-coupled device) EMCCD Andor Technology, Belfast, UK camera. After correction for background fluorescence, determined by addition of ionomycin (1–5 *μ*M) in HBS containing MnCl_2_ (1 mM), fluorescence ratios (F_340_/F_380_) were calibrated to [Ca^2+^]_i_ (Tovey et al., 2003).

#### Measurements of Intracellular cAMP.

Confluent cultures of ASMC grown in 24-well plates and labeled with ^3^H-adenine were incubated under conditions that replicated those used for measurements of [Ca^2+^]_i_. Reactions were terminated by aspiration of medium and addition of ice-cold trichloroacetic acid (5% v/v, 1 ml). After 30 minutes on ice, ^3^H-cAMP was separated from other ^3^H-labeled adenine nucleotides (Pantazaka et al., 2013).

#### Expression of PKI and M3 Muscarinic Receptors.

Plasmids encoding PKI (pRSV-PKI-v2) and its inactive form (pRSV-mut PKI-v2) were from Addgene (cat. no. 45066 and cat. no. 45067; Cambridge, MA) (Day et al., 1989); they were C-terminally tagged with mCherry. Plasmid encoding the human M3 muscarinic acetylcholine receptor was from the cDNA Resource Centre (cat. no. MAR0300000) (Ford et al., 2002). The three constructs were each recombined into BacMam pCMV-DEST. Bacmids were then prepared, and virus was produced from bacmid-infected Sf9 cells according to the manufacturer’s instructions (Thermo Fisher Scientific, Runcorn, UK). ASMC were infected at a multiplicity of infection (MOI) of ∼50 and used after 96 hours.

#### Flash Photolysis of Caged IP_3_.

Confluent cultures of ASMC grown on poly-l-lysine-coated imaging dishes (35-mm diameter with a 7-mm glass insert; MatTek Corporation, Ashland, MA) were loaded (45 minutes, 20°C) with a membrane-permeant form of caged IP_3_ (ci-IP_3_PM, 1 *μ*M) in HBS with probenecid (2.5 mM) and pluronic F127 (0.02% v/v). Fluo-4 AM (4 *μ*M) was then added and after 45 minutes at 20°C, the medium was replaced with HBS containing only probenecid. After a further 45 minutes, this medium was replaced with HBS. Cells were illuminated with a 488-nm diode-based solid-state laser, and emitted fluorescence (500–550 nm) was captured with an EMCCD camera. Three UV flashes (each ∼1-millisecond duration; <345 nm, 3000 *μ*F, 300 V, ∼170 J) from a JML-C2 Xe flash-lamp (Rapp OptoElectronic, Hamburg, Germany) allowed photolysis of caged IP_3_ (ci-IP_3_). Responses are reported as F/F_0_, where F_0_ and F are fluorescence intensities corrected for background recorded from the same region of interest immediately before (F_0_) and after stimulation (F).

#### Measurements of IP_3_ and PLC Activity.

ASMC in 12-well plates were cultured until confluent. The medium was then supplemented with d-*myo*-[2-^3^H]-inositol (10 *μ*Ci/ml) for 48 hours at 37°C. After washing, cells were incubated at 20°C in HBS with LiCl (10 mM) for 5 minutes before stimulation. Reactions were terminated by aspirating medium and adding cold HClO_4_ (1 ml, 0.6 M) containing phytic acid (0.2 mg/ml). After 30 minutes, the acid-extract was removed, the cells were scraped into 50 mM Tris at pH 7 (400 *μ*l), and the pooled extract and cells were centrifuged (10,000*g,* 2 minutes, 4°C). The supernatant was neutralized using K_2_CO_3_ (1 M) with EDTA (5 mM). ^3^H-inositol phosphates were separated using ion-exchange columns.

For assays of IP_3_ mass, ASMC in 75-cm^2^ flasks were stimulated, the medium was removed, and the incubations were terminated by scraping cells into ice-cold ethanol (1 ml). After 30 minutes, extracts were dried and suspended in 300 *μ*l of Tris-EDTA medium (TEM: 50 mM Tris, 1 mM EDTA, pH 8.3). Equilibrium-competition binding using ^3^H-IP_3_ (4.5 nM, 19.3 Ci/mmol), rat cerebellar membranes (10 *μ*g) and cell extract (20–100 *μ*l) in a final volume of 200 *μ*l of TEM was used to determine the IP_3_ content of the extracts (Rossi et al., 2009).

#### Immunoblotting.

Confluent ASMC in 75-cm^2^ flasks or six-well plates were stimulated and then scraped into cold phosphate-buffered saline supplemented with Triton-X-100 (1% w/v), protease inhibitors (one mini-tablet per 10 ml; Roche Applied Science, Burgess Hill, UK), and phosphatase inhibitors (10 *μ*l/ml; Sigma-Aldrich). Scraped cells were disrupted by ∼30 passages through a 28-gauge needle and sonicated (3 × 10 seconds). Proteins were separated by SDS-PAGE (NuPAGE 4%–12% Bis-Tris gels; Invitrogen, Paisley, UK) and transferred to a polyvinylidene difluoride membrane (iBlot; Invitrogen). Membranes were washed (5 minutes) with Tris-buffered saline (TBS: 137 mM NaCl, 20 mM Tris, pH 7.6), blocked by incubation in TBS containing 0.1% Tween-20 (TBS-T) and 5% (w/v) nonfat milk powder (1 hour, 20°C), and then washed with TBS-T (3 × 5 minutes). Blots were incubated for 12 hours at 4°C with primary antibody (1:1000) in TBS-T with 5% (w/v) BSA. After washing (3 × 5 minutes), blots were incubated with horseradish peroxidase-conjugated donkey anti-rabbit secondary antibody (1:2000; Santa Cruz Biotechnology, Dallas, TX) for 1 hour in TBS-T with 5% (w/v) nonfat milk powder. After further washing (3 × 5 minutes), bands were detected using ECL Prime (GE Healthcare, Chalfont St Giles, UK) and quantified using GeneTools (Syngene, Cambridge, UK). The primary rabbit antibodies recognize PKA-phosphorylated sequences RXXS*/T* (AbP1, AbP2, cat. nos. 9621 and 9624; New England Biolabs, Hitchin, UK; * denotes the phosphorylated residue) and vasodilator-stimulated phosphoprotein (VASP [clone 43, BD Biosciences, San Jose, CA]) phosphorylated at Ser-157 (New England Biolabs) or VASP clone 43 (BD Biosciences, San Jose, CA).

#### Quantitative PCR Analysis.

QPCR was performed as described (Tovey et al., 2008) using primers specific for AC subtypes (Ludwig and Seuwen, 2002) and calibrated against expression of the house-keeping gene, glyceraldehyde 3-phosphate dehydrogenase (GAPDH) (Pantazaka et al., 2013). Human BioBank cDNA pooled from a variety of tissues (Primerdesign, Southampton, UK) was used as a positive control for AC subtypes not expressed in ASMC.

#### Statistical Analysis.

Concentration-effect relationships were individually fitted by nonlinear curve-fitting to Hill equations (GraphPad Prism, La Jolla, CA). The absolute sensitivities and amplitudes of the responses to histamine and PGE_2_ varied between patients and with cell passage. Results are, therefore, often presented as normalized values (e.g., as percentages of a maximal response) or as differences between paired comparisons (e.g., *Δ*pIC_50_). Two-tailed paired or unpaired Student’s *t* test, or one-way analysis of variance followed by Bonferroni’s test, were used as appropriate.

## Results

### 

#### Cyclic AMP Mediates Inhibition of Histamine-Evoked Ca^2+^ Signals by PGE_2_.

**Fig. 1.**
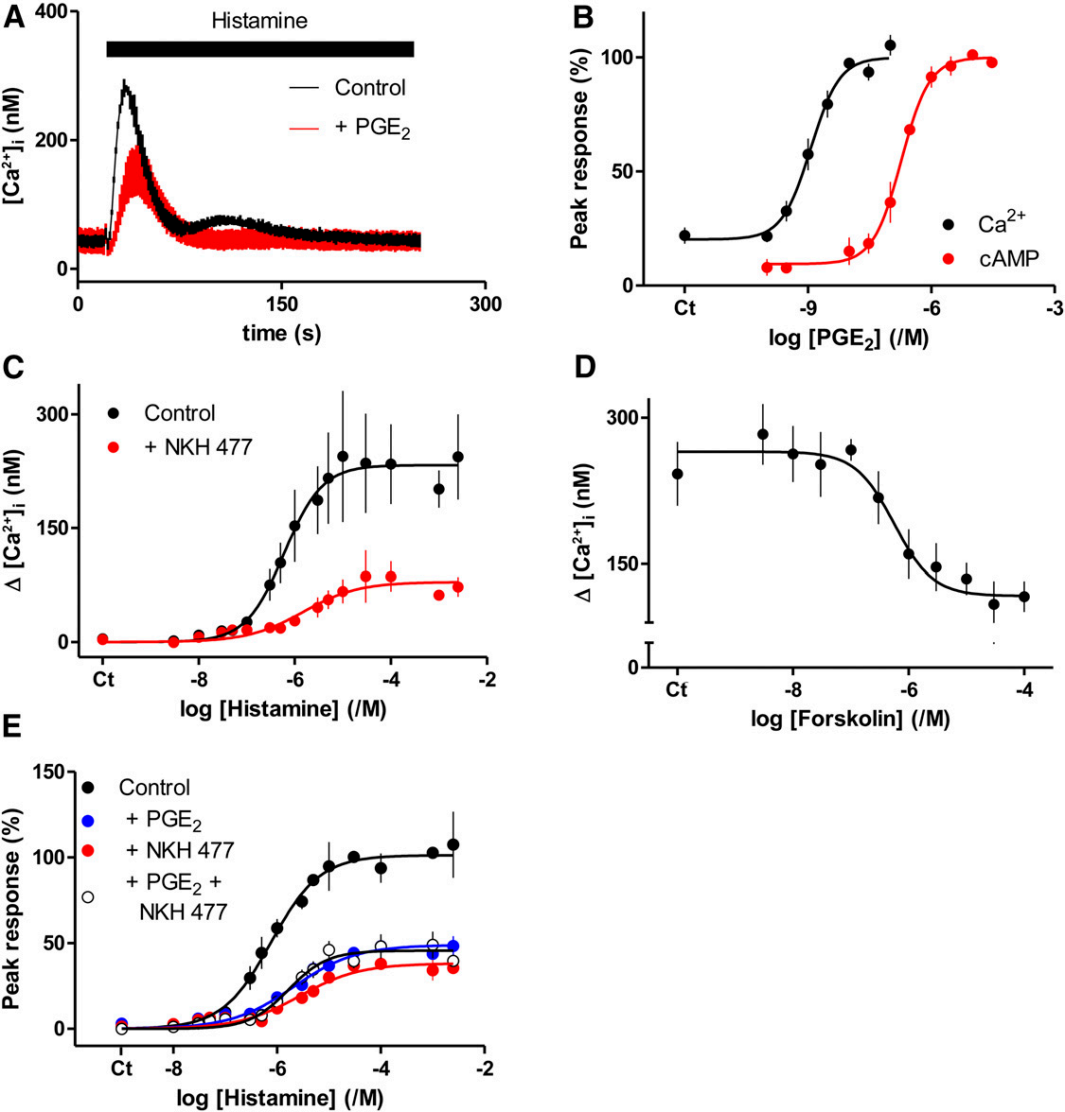
Inhibition of histamine-evoked Ca^2+^ signals by PGE_2_ is mediated by cAMP. (A) Ca^2+^ signals evoked by histamine (3 *μ*M, black bar) alone or with PGE_2_ (10 *μ*M, added 5 minutes before and then during stimulation with histamine). Results show means ± range from two wells on a single plate; they are typical of results from at least four independent experiments. (B) Effects of PGE_2_ on cAMP accumulation (measured after 5 minutes) and inhibition of the peak Ca^2+^ signals evoked by histamine (3 *μ*M). Results, as percentages of maximal inhibition (Ca^2+^) or stimulation (cAMP), are means ± S.E.M. from six and four experiments, respectively. This panel includes some data for cAMP measurements that were published previously (Pantazaka et al., 2013). (C) Effect of NKH 477 (100 *μ*M) on the peak Ca^2+^ signal evoked by histamine. (D) Concentration-dependent effects of forskolin on the peak Ca^2+^ signals evoked by histamine (1 mM). (E) Effect of PGE_2_ (10 *μ*M), NKH 477 (100 *μ*M), or both on the peak Ca^2+^ signals evoked by histamine (as percentages of the maximal response). NKH 477, forskolin, or PGE_2_ were added 5 minutes before and then during stimulation with histamine. Results show means ± S.E.M. from four (C and D) or three (E) independent plates with one to three wells analyzed from each. Ct denotes control.

Figure 1A demonstrates that PGE_2_ inhibits the Ca^2+^ signals evoked by histamine in human ASMC. Most experiments were performed at 20°C to minimize loss of the cytosolic Ca^2+^ indicator. However, in parallel analyses we confirmed that a maximally effective concentration of PGE_2_ (10 *μ*M) caused indistinguishable inhibition of the Ca^2+^ signals evoked by histamine (100 *μ*M) whether the analyses were performed at 20°C (47% ± 12% inhibition, *n* = 4) or 37°C (45% ± 6%). In parallel measurements of the effects of PGE_2_ on intracellular cAMP and histamine-evoked Ca^2+^ signals, the cAMP response [negative logarithm of the half-maximally effective concentration (pEC_50_) = 6.76 ± 0.09, *n* = 4) was ∼140-fold less sensitive to PGE_2_ than were the Ca^2+^ signals [negative logarithm of the half-maximally inhibitory concentration (pIC_50_) = 8.90 ± 0.10, *n* = 6] (Fig. 1B). This relationship is consistent with PGE_2_ evoking formation of more cAMP than needed to maximally inhibit Ca^2+^ signals, and with cAMP lying upstream of the inhibition of Ca^2+^ signaling (Strickland and Loeb, 1981).

Forskolin and its water-soluble analog, NKH 477, directly activate eight of the nine membrane-bound forms of AC (AC1–8) (Seifert et al., 2012). Pretreatment of ASMC with NKH 477 caused a concentration-dependent reduction in both the peak amplitudes of the Ca^2+^ signals evoked by histamine and their sensitivity to histamine (Fig. 1C; Table 1). Similar results were obtained with forskolin (Fig. 1D). Maximally effective concentrations of PGE_2_ and NKH 477 caused indistinguishable inhibition of histamine-evoked Ca^2+^ signals, and their combined maximal effects were not additive (Fig. 1E). These results are consistent with reports showing that forskolin and NKH 477 attenuate the Ca^2+^ signals evoked by receptors, including H_1_ receptors that stimulate PLC in VSM (Yang et al., 1999, and references therein) and other smooth muscles. There has, however, been no prior demonstration that activation of AC inhibits PLC-evoked Ca^2+^ signals in human ASMC.

**TABLE 1:** Inhibition of histamine-evoked Ca^2+^ signals by PGE_2_ and cAMP Cells were incubated for the times shown with the indicated drugs before recording the peak increase in [Ca^2+^]_i_ evoked by histamine (3 *μ*M; 1 mM for forskolin and NKH 477). Results (means ± S.E.M. from *n* independent experiments) show the maximal inhibition of the histamine-evoked Ca^2+^ signals and the pIC_50_ for each drug.

	pIC_50_	Maximal inhibition	*n*
	/M	%	
PGE_2_ (5 min)	9.01 ± 0.05	64 ± 4	9
Butaprost (5 min)	7.28 ± 0.09	61 ± 1	6
L902,688 (5 min)	9.35 ± 0.10	76 ± 4	5
8-Br-cAMP (20 min)	2.98 ± 0.20	85 ± 3	3
6-Bnz-cAMP (20 min)	3.73 ± 0.14	64 ± 5	5
Forskolin (5 min)	6.24 ± 0.11	57 ± 6	4
NKH 477 (5 min)	5.50 ± 0.18	53 ± 4	5

**Fig. 2.**
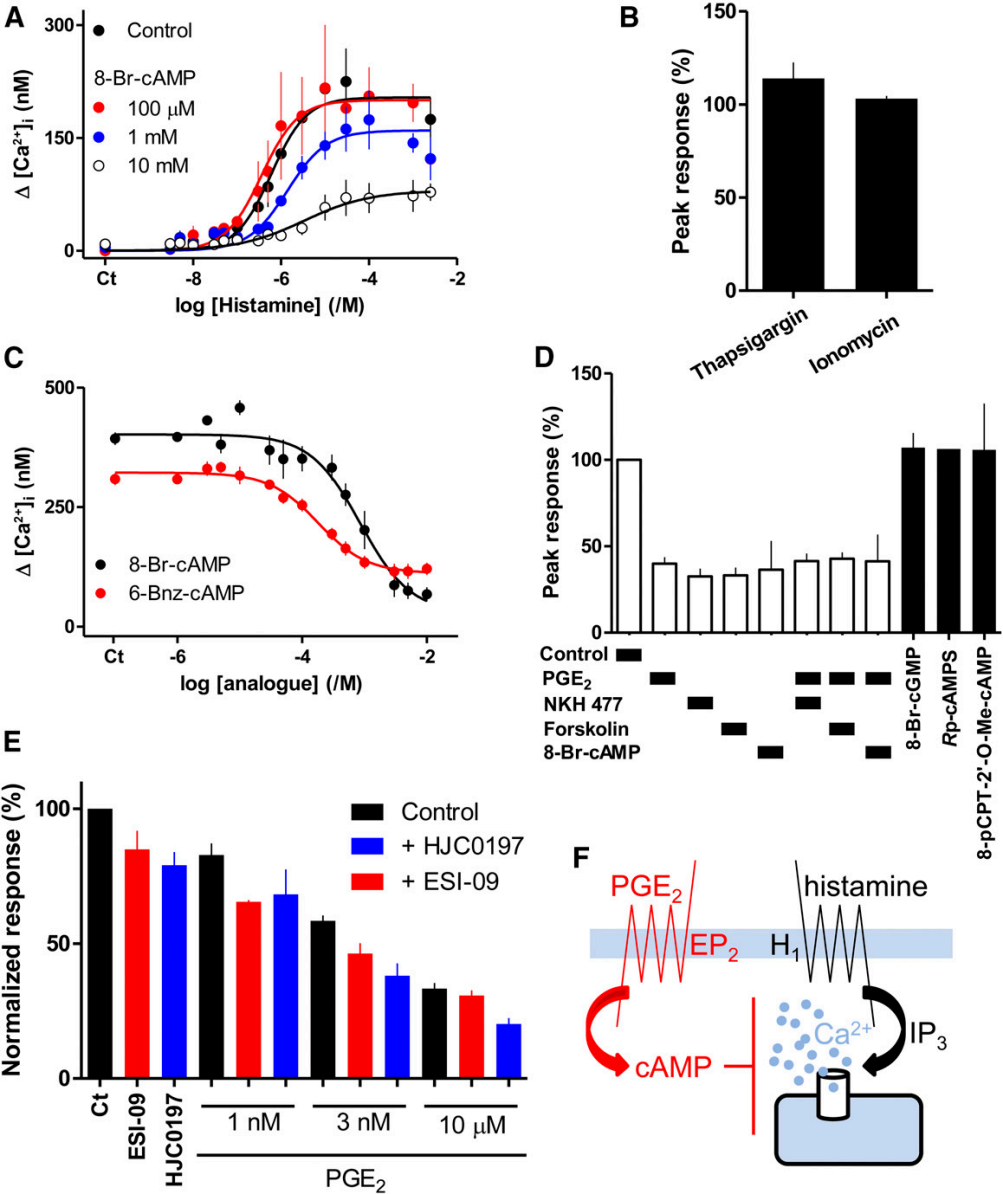
Cyclic AMP mediates inhibition of histamine-evoked Ca^2+^ signals by PGE_2_. (A) Effect of 8-Br-cAMP (added 20 minutes before histamine) on the peak Ca^2+^ signals evoked by the indicated concentrations of histamine. Results are means ± S.E.M. from at least three experiments with one to three wells in each. (B) 8-Br-cAMP (10 mM, 20 minutes) had no effect on the Ca^2+^ content of the intracellular stores as revealed by the increases in [Ca^2+^]_i_ evoked by addition of thapsigargin (1 *μ*M) or ionomycin (1 *μ*Μ) in Ca^2+^-free HBS. Results (percentages of responses without 8-Br-cAMP) are means ± S.E.M. from five experiments with three to four wells analyzed in each. (C) Effect of the indicated cyclic nucleotides (added 20 minutes before histamine) on the peak Ca^2+^ signals evoked by histamine (3 *μ*M). Results are means ± S.E.M. from three to five experiments with two to three wells in each. (D) Effect of NKH 477 (100 *μ*M, 5 minutes), forskolin (100 *μ*M, 5 minutes), 8-Br-cAMP (10 mM, 20 minutes), PGE_2_ (10 *μ*Μ, 5 minutes), 8-Br-cGMP (10 mM, 20 minutes), *R*p-cAMPS (10 mM, 20 minutes), or 8-pCPT-2*′*-O-Me-cAMP (10 mM, 20 minutes) alone or in combination on the peak Ca^2+^ signals evoked by histamine (1 mM). Results (as percentages of the response to histamine alone) are means ± S.E.M. from three experiments with two to three wells in each. Results for *R*p-cAMPS are from a single experiment with three replicates, limited by the availability of this expensive analog. (E) Effects of the EPAC antagonists, ESI-09 and HJC0197 (10 *μ*M, 20 minutes), on the Ca^2+^ signals evoked by histamine (3 *μ*M) added 5 minutes before and then during treatment with the indicated concentrations of PGE_2_. Results are expressed as percentages of the paired response to histamine alone (means ± S.E.M., *n* = 3–5; *n* = 2 for the antagonists with 1 and 3 nM PGE_2_, where error bars show ranges). (F) The results establish that cAMP mediates inhibition of histamine-evoked Ca^2+^ signals by PGE_2_.

High concentrations of 8-Br-cAMP also inhibited histamine-evoked Ca^2+^ signals by reducing the maximal response and the sensitivity to histamine (Fig. 2A). 8-Br-cAMP had no effect on the Ca^2+^ content of the intracellular stores (Fig. 2B). The effects of 8-Br-cAMP were mimicked by *S*p-cAMPS, which activates PKA and EPACs, and by 6-Bnz-cAMP and 8-CPT-6-Phe-cAMP, which activate PKA but not EPACs (Fig. 2C; Supplemental Fig. S1; and Table 1). A high concentration (10 mM) of 8-pCPT-2′-O-Me-cAMP, a membrane-permeant activator of EPACs; and *R*p-cAMPS and *R*p-8-CPT-cAMPS, antagonists of both PKA and EPACs, were ineffective (Fig. 2D; Supplemental Fig. S1).

The negative result with 8-pCPT-2′-O-Me-cAMP is important because this analog is more membrane-permeable than 8-Br-cAMP, and it both binds with greater affinity than cAMP to EPACs and more effectively activates them (Gloerich and Bos, 2010). Furthermore, antagonists of EPACs 1 and 2, HJC0197 (Chen et al., 2012), and ESI-09 (Almahariq et al., 2013) (10 *μ*M, 20 minutes) did not prevent the inhibition of histamine-evoked Ca^2+^ signals by PGE_2_ (Fig. 2E). Higher concentrations (50 *μ*M) of either antagonist abolished the Ca^2+^ signals evoked by histamine (data not shown). We have not explored this effect further, although the antagonists caused similar inhibition of carbachol-evoked Ca^2+^ signals in human embryonic kidney 293 cells (Meena et al., 2015). Others have also reported nonspecific effects of these EPAC antagonists (Rehmann, 2013).

Maximally effective concentrations of PGE_2_, forskolin, NKH 477, or 8-Br-cAMP similarly attenuated the Ca^2+^ signals evoked by histamine, and combinations of the treatments were not additive (Fig. 2D). These results establish that inhibition of histamine-evoked Ca^2+^ signals by PGE_2_ is mediated by cAMP (Fig. 2F) and does not require EPACs.

#### Inhibition of Histamine-Evoked Ca^2+^ Signals by PGE_2_ is Not Mediated by cGMP-Dependent Protein Kinase.

**Fig. 3.**
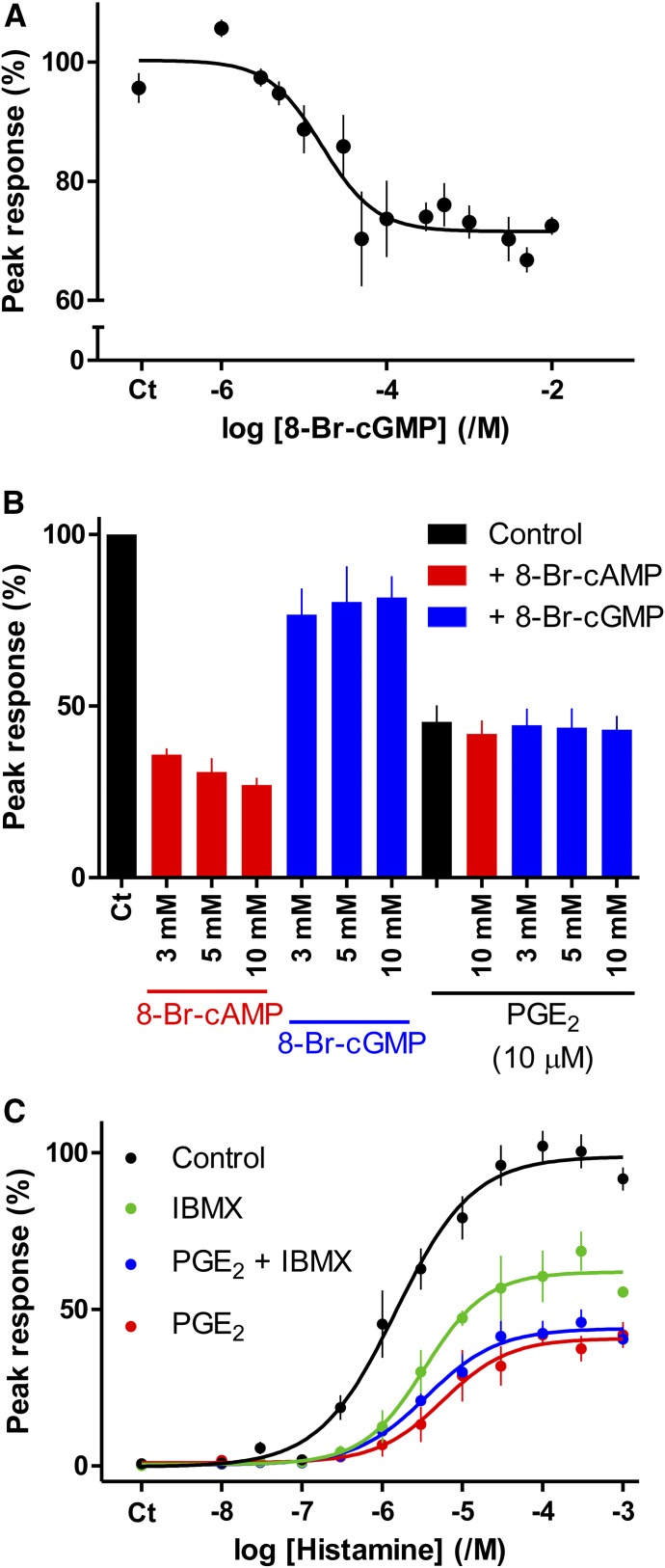
Inhibition of histamine-evoked Ca^2+^ signals by PGE_2_ is not mediated by cGMP. (A) Concentration-dependent effects of 8-Br-cGMP (20 minutes) on the peak Ca^2+^ signals evoked by histamine (3 *μ*M). (B) Similar experiments show the responses to histamine (3 *μ*M) after preincubation with PGE_2_ (10 *μ*M, 5 minutes) and/or the indicated concentrations of 8-Br-cAMP or 8-Br-cGMP (20 minutes). (C) Peak Ca^2+^ signals evoked by histamine alone or after incubation with IBMX (1 mM, 20 minutes), PGE_2_ (10 *μ*M, 5 minutes), or both. Results (as percentages of response to histamine alone) are means ± S.E.M. from five (A), three to six (B; *n* = 2 for 3 and 5 mM 8-Br-cAMP, where ranges are shown), and four to seven (C) independent experiments with two to three wells in each.

Cyclic AMP may directly activate PKG in arterial smooth muscle, although this is contentious [see references in Morgan et al. (2014)]. Cyclic AMP could, however, increase the concentration of cGMP by competing with it for degradation by cyclic nucleotide phosphodiesterases (PDEs). Stimulation of PKG might then attenuate IP_3_-evoked Ca^2+^ release (Masuda et al., 2010). There is evidence, however, that expression of proteins involved in PKG signaling in VSM are downregulated in culture (Lincoln et al., 2006, and references therein). 8-Br-cGMP (pIC_50_ = 4.50 ± 0.29, *n* = 5) partially inhibited Ca^2+^ signals evoked by a submaximal concentration of histamine, but the maximal inhibition was less than half that evoked by PGE_2_ or 8-Br-cAMP (Fig. 3, A and B). Furthermore, and in contrast to the effects of 8-Br-cAMP (Fig. 2C), 8-Br-cGMP did not inhibit the Ca^2+^ signals evoked by a maximal histamine concentration (Fig. 2D). Prolonged incubation with IBMX (20 minutes, 1 mM), a nonselective inhibitor of PDEs, inhibited histamine-evoked Ca^2+^ signals, but the inhibition (33% ± 3%, *n* = 4) was less than that caused by PGE_2_ (56% ± 3%) (Fig. 3C). More importantly, a maximal concentration of PGE_2_ similarly inhibited histamine-evoked Ca^2+^ signals in the presence and absence of IBMX (Fig. 3C), demonstrating that the effects of PGE_2_ are not mediated by inhibition of PDEs. We conclude that inhibition of histamine-evoked Ca^2+^ signals by PGE_2_ is not mediated by inhibition of PDEs and consequent accumulation of cGMP.

#### Histamine and 8-Br-cAMP Stimulate PKA-Mediated Protein Phosphorylation in Different Microenvironments.

Most effects of cAMP are mediated by PKA, EPACs, or cyclic nucleotide-regulated plasma membrane cation channels (Gloerich and Bos, 2010; Cooper and Tabbasum, 2014). The latter cannot mediate the effects of cAMP on IP_3_-evoked Ca^2+^ release, nor are EPACs responsible. We therefore assessed the role of PKA.

**Fig. 4.**
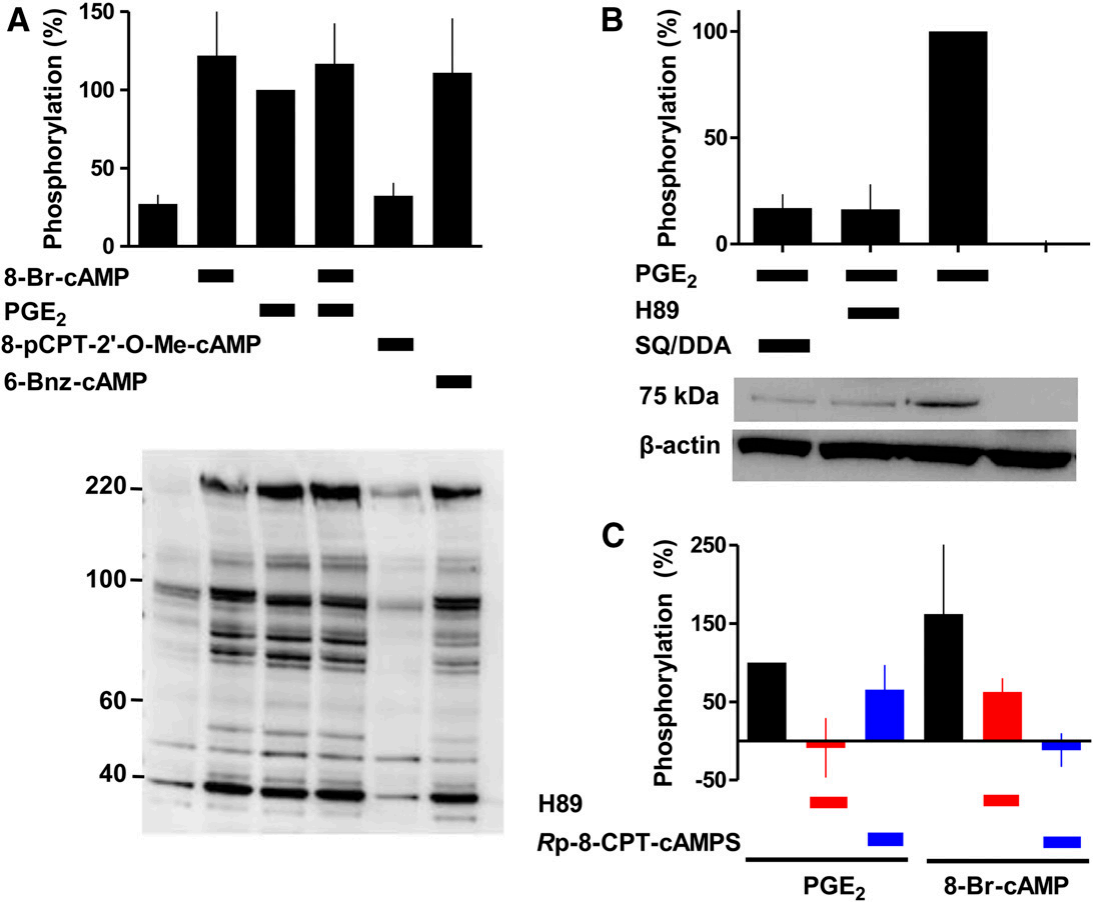
PGE_2_ and 8-Br-cAMP stimulate PKA-mediated protein phosphorylation. (A) Typical immunoblot using AbP2 (see *Materials and* *Methods*) showing PKA-phosphorylated proteins from cells stimulated with cAMP analogs (10 mM, 20 minutes) or PGE_2_ (10 *μ*M, 5 minutes). Summary results (means ± S.D., *n* = 3–4) show the band intensity across the entire gel as a percentage of that from cells stimulated with PGE_2_. M_r_ markers (kilodaltons) shown alongside gel. (B) Similar analyses using AbP1 show the effects of H89 (10 *μ*M, 10 minutes) or SQ/DDA (1 mM SQ22536 and 200 *μ*M DDA, 20 minutes) on the phosphorylation of a 75-kDa band in response to PGE_2_ (10 *μ*M. 5 minutes). Summary results (means ± S.D., *n* = 3–5) show band intensities as percentages of the matched response to PGE_2_ alone. (C) Similar analysis to (A), for cells stimulated with PGE_2_ (10 *μ*M, 5 minutes) or 8-Br-cAMP (10 mM, 20 minutes) alone or after incubation (20 minutes) with *R*p-8-CPT-cAMPS (1 mM) H89 (10 *μ*M). Results (means ± S.D., *n* = 3–4) show the increase in phosphorylation evoked by the indicated stimuli expressed as a percentage of the matched response to PGE_2_ alone.

Immunoblotting with an antiserum that recognizes sequences phosphorylated by PKA showed that maximally effective concentrations of PGE_2_ or 8-Br-cAMP stimulated similar levels of protein phosphorylation in ASMC and their effects were nonadditive (Fig. 4A). The phosphorylation was mimicked by 6-Bnz-cAMP but not by the EPAC-selective analog 8-pCPT-2*′*-O-Me-cAMP (Fig. 4A). PGE_2_-evoked protein phosphorylation was attenuated by inhibition of either PKA (with H89) or AC [with 1 mM SQ 22536 with 200 *μ*M DDA (SQ/DDA)] (Fig. 4B).

Maximal concentrations of PGE_2_ and 8-Br-cAMP caused phosphorylation of the same proteins (Fig. 4A), but the two stimuli differed in their susceptibility to PKA inhibitors. *R*p-8-CPT-cAMPS, an inhibitor of PKA that competes with cAMP by binding to the regulatory subunit of PKA, abolished the phosphorylation evoked by 8-Br-cAMP but only partially inhibited that evoked by PGE_2_ (Fig. 4C). Conversely, H89, which inhibits PKA (and other kinases) by competing for the ATP-binding site, abolished the phosphorylation evoked by PGE_2_ but caused lesser inhibition of the response to 8-Br-cAMP (Fig. 4C). Similar results were obtained when an antiserum to phospho-VASP was used to assess PKA-mediated phosphorylation (Supplemental Fig. S2).

These results suggest that PKA activated by PGE_2_ may be exposed to high local concentrations of cAMP, which might then effectively compete with the inhibitor *R*p-8-CPT-cAMPS. Conversely, PKA activated by 8-Br-cAMP, which would probably be uniformly distributed within the cell, may be more accessible to ATP than PKA activated by PGE_2_, and so less susceptible to inhibition by H89.

#### Inhibition of Histamine-Evoked Ca^2+^ Signals by PGE_2_ Is Attenuated by Inhibition of PKA.

**Fig. 5.**
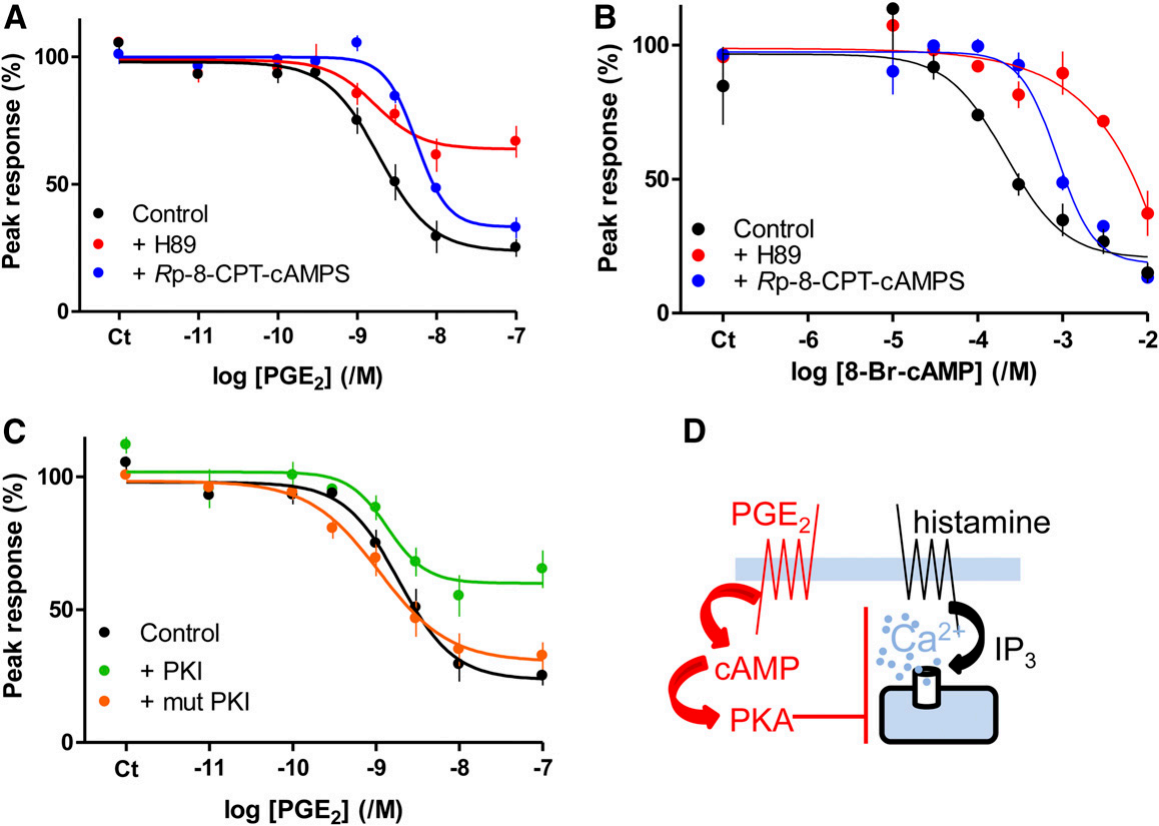
Inhibition of histamine-evoked Ca^2+^ signals by PGE_2_ or 8-Br-cAMP requires PKA. (A and B) Effects of PGE_2_ (5 minutes) or 8-Br-cAMP (20 minutes) on the peak Ca^2+^ signals evoked by histamine (3 *μ*M) alone or with H89 (10 *μ*M, 20 minutes) or *R*p-8-CPT-cAMPS (1 mM, 20 minutes). (C) Effects of PGE_2_ (5 minutes) on the Ca^2+^ signals evoked by histamine (3 *μ*M) in cells infected with baculovirus expressing PKI or its inactive form (mut PKI). Results (A–C) are means ± S.E.M. from three experiments with two to three wells in each. (D) The results suggest that PKA mediates the inhibition of histamine-evoked Ca^2+^ signals by PGE_2_.

Inhibition of histamine-evoked Ca^2+^ signals by PGE_2_ or 8-Br-cAMP was inhibited by *R*p-8-CPT-cAMPS (1 mM), which reduced the sensitivity to PGE_2_ (decreasing the pIC_50_ for PGE_2_ from 8.68 ± 0.17 to 8.26 ± 0.05, *n* = 3; *Δ*pIC_50_ = 0.4 ± 0.1, where *Δ*pIC_50_ = pIC_50_^control^ – pIC_50_*^R^*^p-8-CPT-cAMPS^) and 8-Br-cAMP (*Δ*pIC_50_ = 0.6 ± 0.1) without affecting the maximal inhibition (Fig. 5, A and B). These results are consistent with *R*p-8-CPT-cAMPS competitively inhibiting cAMP binding to PKA and thereby attenuating the effects of cAMP on Ca^2+^ signals.

H89 (10 *μ*M) also attenuated the inhibition of histamine-evoked Ca^2+^ signals by 8-Br-cAMP (*Δ*pIC_50_ = 1.13 ± 0.18 *n* = 3) and PGE_2_ (Fig. 5, A and B). In keeping with our analyses of protein phosphorylation (Fig. 4C), the inhibition of histamine-evoked Ca^2+^ signals by maximal concentrations of PGE_2_ were less effectively inhibited by H89 than were the effects of maximal concentrations of 8-Br-cAMP (compare Fig. 5, A and B).

PKI inhibits PKA by competing with its peptide substrates. We could not achieve effective inhibition of PKA-mediated protein phosphorylation with myristoylated-PKI (10 *μ*M, 20 minutes, data not shown). But using a baculovirus, we achieved expression of PKI in >90% of cells, and this caused 49% ± 6% (*n* = 3) inhibition of the VASP phosphorylation evoked by PGE_2_ (100 nM) (Supplemental Fig. S3). Expression of an inactive PKI (mut PKI) had no effect on PGE_2_-evoked protein phosphorylation (Supplemental Fig. S3). The effects of H89 and PKI on the inhibition of histamine-evoked Ca^2+^ signals by PGE_2_ were similar: Each substantially reduced the maximal inhibition without significantly affecting the IC_50_ for PGE_2_ (Fig. 5, A and C). The effects of H89 on inhibition of histamine-evoked Ca^2+^ signals by selective agonists of EP_2_ (butaprost) and EP_4_ (L902,688) receptors were similar to those observed with PGE_2_ (Supplemental Fig. S4). We conclude that inhibition of histamine-evoked Ca^2+^ signals by PGE_2_ is mediated by cAMP and requires PKA (Fig. 5D).

#### PGE_2_ Does Not Inhibit Ca^2+^ Release Evoked by Direct Activation of IP_3_Rs.

The rate at which [Ca^2+^]_i_ recovered from the peak Ca^2+^ signal evoked by histamine was unaffected by PGE_2_ (half-times for recovery were 19 ± 1 and 17 ± 1 seconds, after histamine alone or with PGE_2_, respectively; *n* = 11) (Supplemental Fig. S5). This suggests that the attenuated Ca^2+^ signals do not result from PGE_2_ stimulating Ca^2+^ extrusion from the cytosol.

**Fig. 6.**
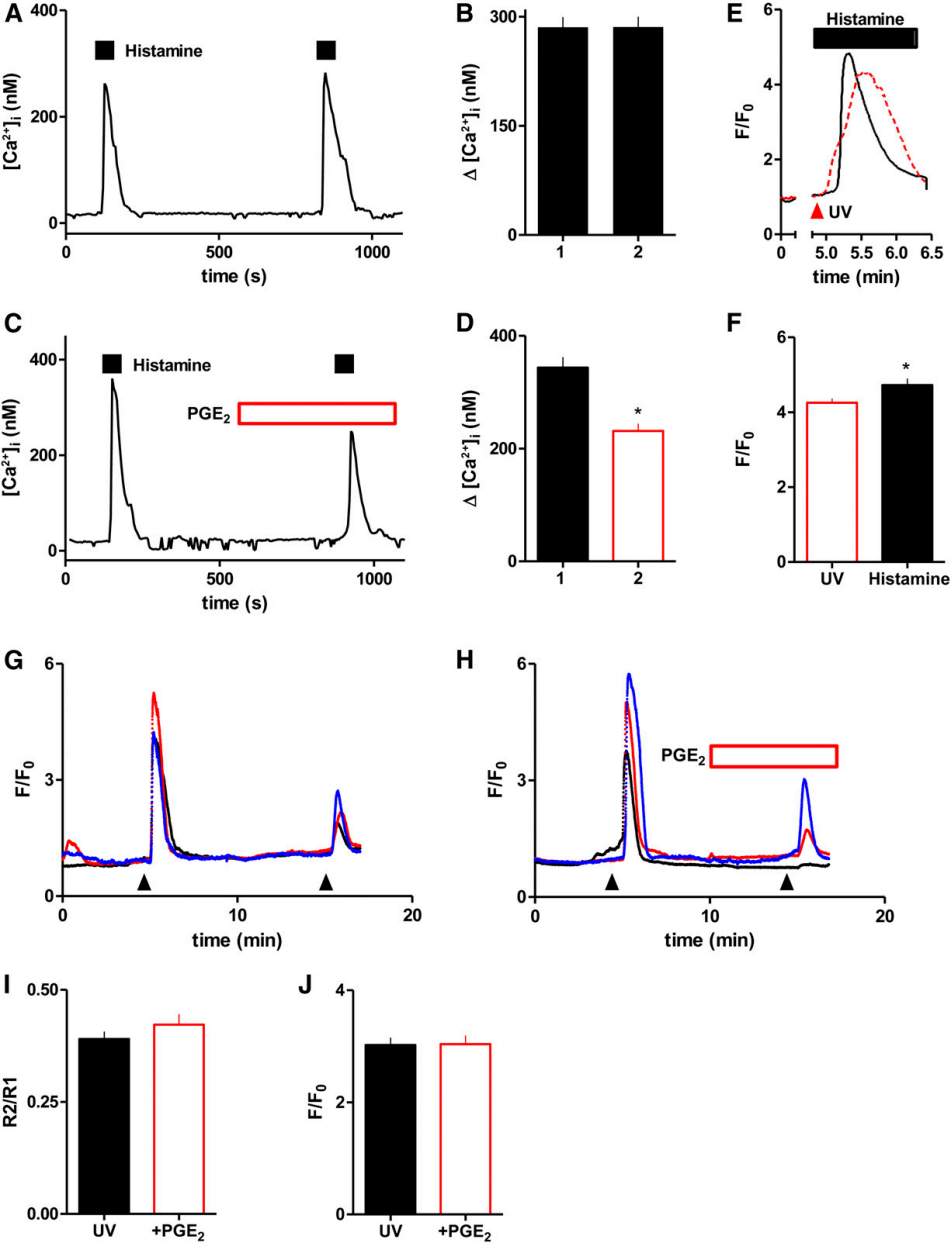
PGE_2_ does not inhibit Ca^2+^ signals evoked by direct activation of IP_3_ receptors. (A–D) Typical fluorescence traces from single Fura-2-loaded ASMC sequentially stimulated with histamine (1 mM) alone (A) or with PGE_2_ (10 *μ*M) (C). Summary results (means ± S.E.M., 65 cells from six independent fields) show peak amplitudes of the first and second responses to histamine in the absence (B) or presence (D) of PGE_2_. (E) Typical fluorescence traces from single Fluo-4-loaded ASMC stimulated with a UV flash to photolyse ci-IP_3_ (red trace) or histamine (1 mM, black trace). (F) Summary results (means ± S.E.M. from 35 and 118 cells, for histamine and ci-IP_3_ respectively) show peak responses. (G and H) Three typical fluorescence traces from single ASMC stimulated twice with UV flashes (arrowheads) alone (G) or with PGE_2_ (10 *μ*M) (H). (I) Summary results (means ± S.E.M. from 50–68 cells) show relative amplitudes of the peak responses (R2/R1). (J) Summary results (means ± S.E.M. from 36–43 cells) show F/F_0_ (see *Materials and* *Methods*) for cells stimulated with a UV flash alone or with PGE_2_ (10 *μ*M added 5 minutes before flash). *P < 0.05, relative to the response to histamine alone (D, paired Student’s *t* test) or to the UV flash alone (F, unpaired Student’s *t* test).

We used flash-photolysis of ci-IP_3_ to activate IP_3_R directly in Fluo-4-loaded ASMC. Single-cell analyses of ASMC established that most cells (99% ± 1%, from 12 fields) responded to histamine (1 mM) with an increase in [Ca^2+^]_i_, and that two successive challenges with histamine evoked indistinguishable Ca^2+^ signals (Fig. 6, A and B). PGE_2_ reduced the peak amplitude of the Ca^2+^ signal evoked by a second histamine challenge by 28% ± 4% (*n* = 65 cells), without significantly affecting the number of cells that responded (91% ± 8% and 83% ± 7% for control and PGE_2_-treated cells, respectively) (Fig. 6, C and D). These results confirm that under the conditions used for uncaging ci-IP_3_, PGE_2_ inhibits histamine-evoked Ca^2+^ signals.

ASMC loaded with ci-IP_3_ responded to UV flashes with rapid increases in Fluo-4 fluorescence (F/F_0_, see *Materials and* *Methods*). The amplitudes of these signals were less than those evoked by a maximal concentration of histamine (Fig. 6, E and F), confirming that responses to photolysis of ci-IP_3_ were not saturated. Although cells responded similarly to successive histamine challenges (Fig. 6, A and B), the response to a second photolysis of ci-IP_3_ was smaller than the first (Fig. 6G), presumably because each stimulus depleted a fraction of the ci-IP_3_. We therefore used two methods to assess the effects of PGE_2_ on the Ca^2+^ signals evoked by photolysis of ci-IP_3_. Cells were either stimulated twice with a UV stimulus, and the amplitude of the second response (with or without PGE_2_) was compared with the first response for each cell (R2/R1) (Fig. 6, G–I), or cells were stimulated once with UV flashes alone or in the presence of PGE_2_ (Fig. 6J). Both analyses concur in demonstrating that PGE_2_ has no significant effect on the Ca^2+^ signals evoked by IP_3_ (Fig. 6, G–J). The results with ci-IP_3_ therefore demonstrate that PGE_2_ does not affect the interactions of IP_3_ with IP_3_R. Furthermore, because the peak IP_3_-evoked Ca^2+^ signals were unaffected by PGE_2_ under conditions where it attenuates responses to histamine (Fig. 6, I and J), the results provide additional evidence that PGE_2_ does not stimulate Ca^2+^ removal from the cytosol.

The product of ci-IP_3_ photolysis is an active but modified form of IP_3_ (d-2,3-*O*-isopropylidene-*myo*-inositol 1,4,5-trisphosphate) (Dakin and Li, 2007) that is not a substrate for IP_3_ 3-kinase and may differ from IP_3_ in its rate of dephosphorylation. Our results do not therefore exclude the possibility that PGE_2_ may accelerate degradation of IP_3_. These results suggest that PGE_2_ attenuates histamine-evoked Ca^2+^ signals by inhibiting IP_3_ formation, stimulating IP_3_ degradation, and/or disrupting IP_3_ delivery to IP_3_Rs.

#### PGE_2_ Attenuates Histamine-Evoked Accumulation of IP_3_.

**Fig. 7.**
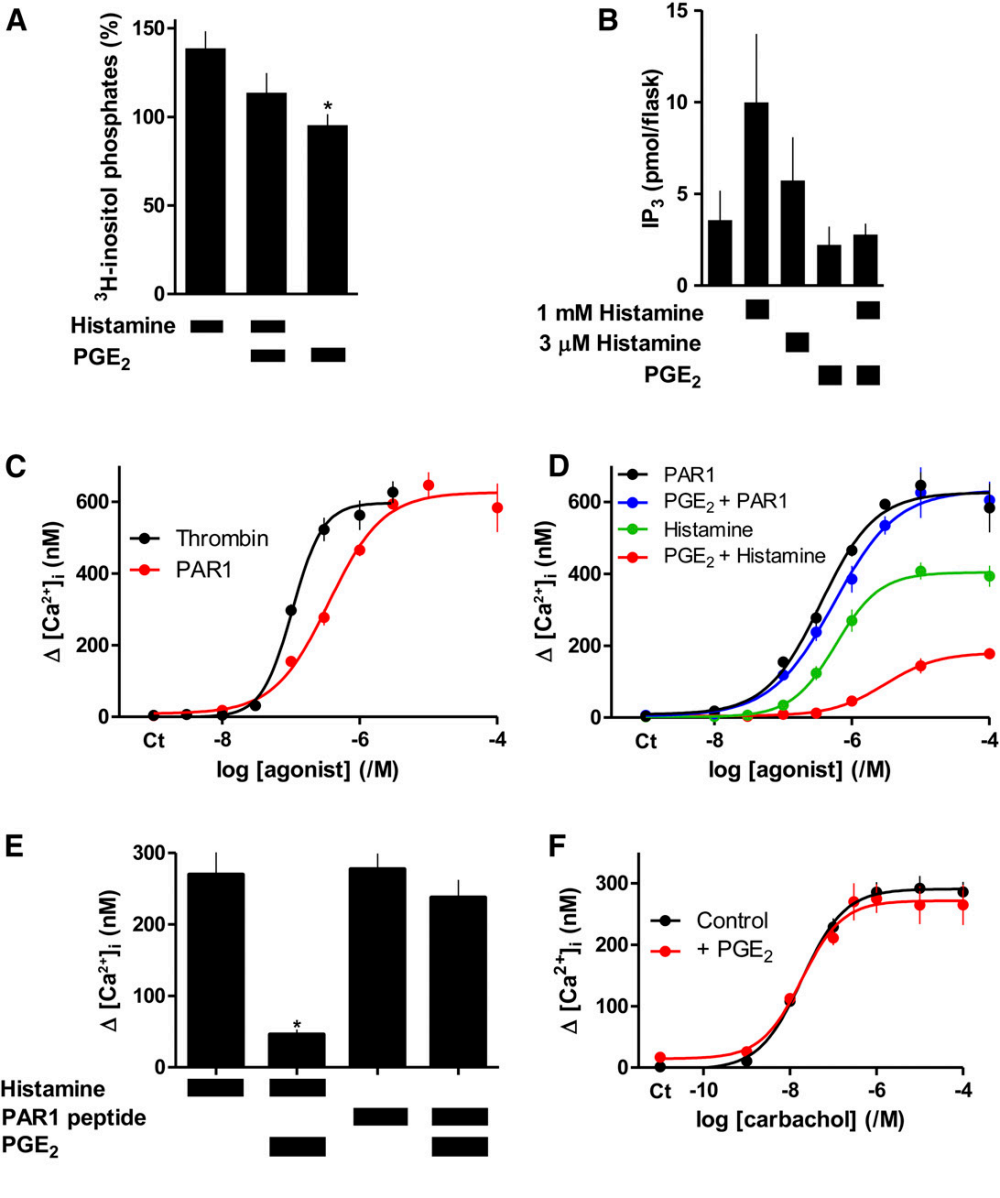
PGE_2_ selectively attenuates activation of PLC by histamine. (A) Accumulation of ^3^H-inositol phosphates (30 minutes) in cells incubated with LiCl and stimulated with histamine (1 mM) alone or with PGE_2_ (10 *μ*M). Results are shown as percentages of matched analyses from unstimulated cells. (B) Mass assays of (1,4,5)IP_3_ from cells treated with histamine (1 minute) alone or after treatment with PGE_2_ (10 *μ*M, 5 minutes). Results (A and B) are means ± S.E.M., *n* = 3–5, *P < 0.05, relative to 1 mM histamine, one-way analysis of variance with Bonferroni’s test. (C) Peak Ca^2+^ signals evoked by the indicated concentrations of thrombin or PAR1 peptide. (D) Effects of PGE_2_ (10 *μ*M, 5 minutes) on the peak Ca^2+^ signals evoked by the indicated concentrations of histamine or PAR1 peptide. Results (C and D) are means ± S.E.M., *n* = 4–5. (E) Comparison of the effects of PGE_2_ on Ca^2+^ signals of similar amplitude evoked by histamine or PAR1 peptide. (F) Peak increases in [Ca^2+^]_i_ in ASMC heterologously expressing M3 muscarinic receptors and stimulated in Ca^2+^-free HBS with the indicated concentrations of carbachol alone or with PGE_2_ (10 *μ*M, 5 minutes). Results are means ± S.E.M., *n* = 3, with six replicates for each. There was no response to carbachol in normal ASMC (data not shown).

Using an assay that reports PLC activity (stimulation after blocking inositol monophosphate degradation by Li^+^), histamine (1 mM, 30 minutes) stimulated a small accumulation of ^3^H-inositol phosphates in ASMC. Although the response was modestly attenuated by PGE_2_ (10 *μ*M), the effect was not statistically significant (Fig. 7A). Using an IP_3_R-based bioassay that detects only (1,4,5)IP_3_, histamine stimulated IP_3_ accumulation, and the response was attenuated by PGE_2_, although the latter again failed to achieve statistical significance (Fig. 7B). We also attempted to measure histamine-evoked IP_3_ formation in single cells using a fluorescence resonance energy transfer (FRET)-based IP_3_ sensor (Gulyas et al., 2015), but the signals were too small to resolve reliably any inhibitory effect of PGE_2_. Available genetically encoded IP_3_ sensors are known to have limited dynamic range and limited capacity to resolve small changes in intracellular IP_3_ concentration (Miyamoto and Mikoshiba, 2017).

We assessed the responses of ASMC to other stimuli (ATP, bradykinin, carbachol, phenylephrine, and thrombin) that might be expected to evoked Ca^2+^ signals through receptors that stimulate Gq (results not shown). Only thrombin reproducibly evoked substantial increases in [Ca^2+^]_i_. Thrombin is a protease that cleaves the type 1 protease-activated receptor (PAR1) to unmask an N-terminal ligand. Thrombin and the PAR1 peptide itself evoked concentration-dependent increases in [Ca^2+^]_i_ in ASMC (Fig. 7C). In parallel analyses, PGE_2_ (10 *μ*M, 5 minutes) attenuated the Ca^2+^ signals evoked by histamine without affecting those evoked by PAR1 peptide (Fig. 7D). Although the maximal increase in [Ca^2+^]_i_ evoked by PAR1 peptide was larger than that evoked by histamine, with concentrations of histamine and the PAR1 peptide that evoked comparable increases in [Ca^2+^]_i_, only the response to histamine was inhibited by PGE_2_ (Fig. 7E). After heterologous expression of human muscarinic M3 acetylcholine receptors in ASMC, carbachol evoked a concentration-dependent (pEC_50_ = 7.72 ± 0.04, *n* = 3) increase in [Ca^2+^]_i_, with a maximal increase (272 ± 31 nM, *n* = 3) comparable to that evoked by histamine (204 ± 13 nM, Fig. 2A). However, the responses to carbachol were unaffected by PGE_2_ (Fig. 7F). These results, demonstrating that PGE_2_ selectively inhibits the Ca^2+^ signals evoked by histamine, suggest that the inhibition probably does not arise downstream of PLC.

#### Histamine-Evoked Ca^2+^ Signals Are Inhibited by Local cAMP Signals.

**Fig. 8.**
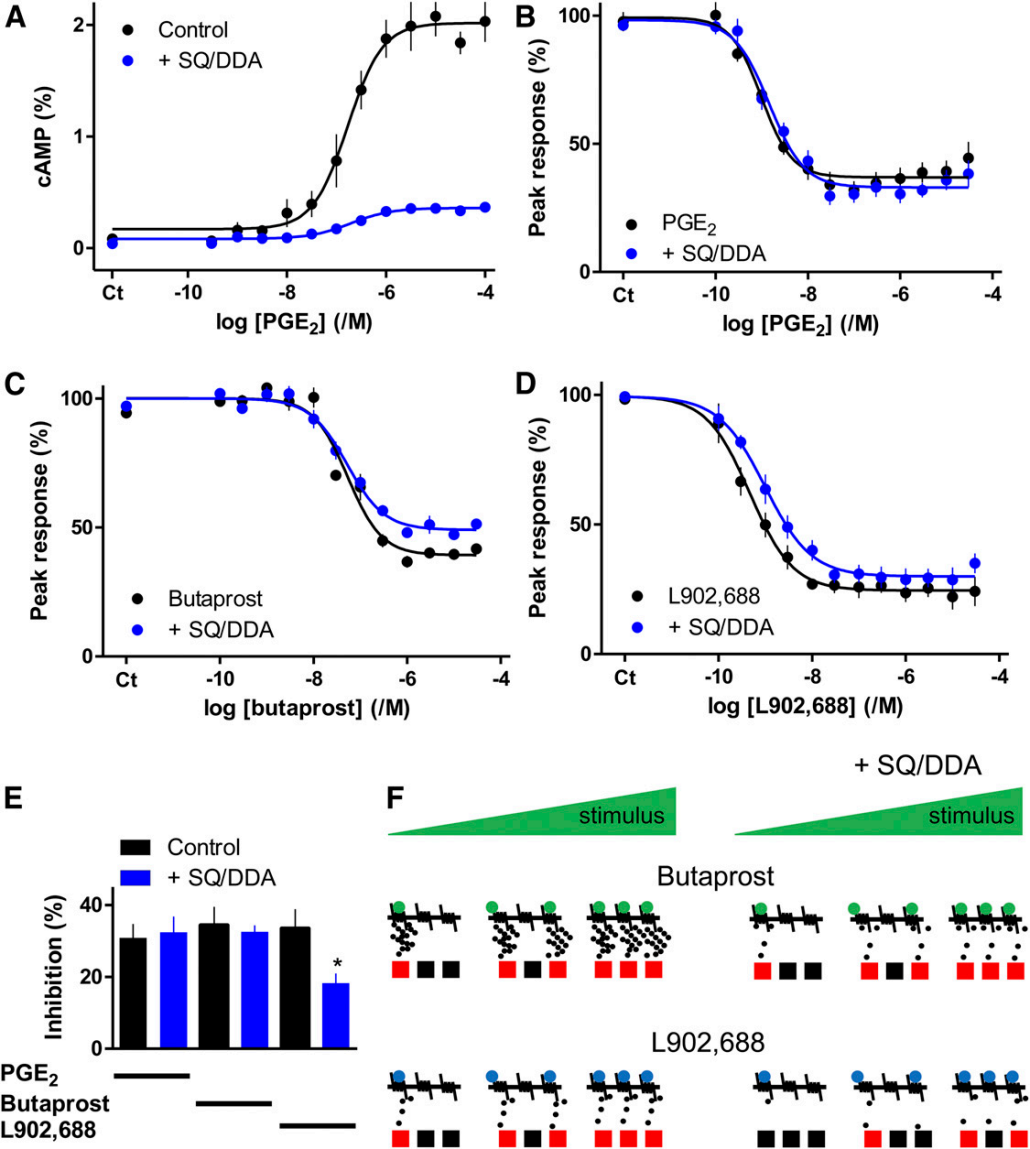
Signaling from EP receptors to Ca^2+^ signals via cAMP junctions. (A) Concentration-dependent effects of PGE_2_ on intracellular cAMP (5 minutes) alone or after treatment with SQ/DDA (20 minutes). Results [cAMP/(ATP + ADP + cAMP), %] are means ± S.E.M. from four experiments [three of these were published in Pantazaka et al. (2013)]. (B–D) Concentration-dependent effects of PGE_2_, butaprost, or L902,688 on the Ca^2+^ signals evoked by histamine (3 *μ*M) in the presence of SQ/DDA (20 minutes). Results are means ± S.E.M. from nine (B), six (C), or five (D) experiments with two to three wells in each. (E) Summary data show the effects of SQ/DDA on the inhibition of histamine-evoked Ca^2+^ signals by concentrations of PGE_2_ (1 nM), butaprost (30 nM), and L902,688 (0.3 nM) that approximately match their IC_50_ values. (F) Junctional delivery of cAMP to the PKA (squares) through which it inhibits (red) histamine-evoked Ca^2+^ signals. Activation of EP_2_ or EP_4_ receptors with butaprost or L902,688, respectively, inhibits histamine-evoked Ca^2+^ signals, but the latter evokes formation of less cAMP. We propose, from our results with SQ/DDA, that cAMP is locally delivered to PKA within each signaling junction at a concentration more than sufficient to cause a maximal effect. The concentration-dependent inhibition of Ca^2+^ signals by prostanoids is proposed to result from recruitment of all-or-nothing cAMP signaling junctions, rather than from graded increases in activity within individual junctions. EP_2_ receptors deliver more cAMP than EP_4_ receptors and are therefore more resistant to inhibition of AC by SQ/DDA. Hence inhibition of Ca^2+^ signals by EP_4_ receptors is partially inhibited by SQ/DDA, whereas the response to EP_2_ receptors is insensitive (right). *P < 0.05, paired Student's *t*-test relative to control.

Inhibitors of AC (SQ/DDA) attenuated PGE_2_-evoked cAMP formation (by 79% ± 2%, *n* = 4) (Fig. 8A) and protein phosphorylation (Fig. 4B). However, SQ/DDA had no effect on the inhibition of histamine-evoked Ca^2+^ signals evoked by PGE_2_ or butaprost (Fig. 8, B and C). Although cAMP mediates the inhibition of Ca^2+^ signals by PGE_2_, the response to a maximal concentration of PGE_2_ might survive substantial inhibition of AC because it stimulates formation of more cAMP than needed to maximally inhibit Ca^2+^ signals (Fig. 1B). However, the same argument cannot account for the lack of effect of SQ/DDA on responses to submaximal concentrations of PGE_2_. How might a submaximal response to PGE_2_ be unaffected by substantial inhibition of cAMP formation and PKA activity (Fig. 4B; Fig. 8, A and B)?

A possible explanation is that SQ 22356 and DDA, related inhibitors that bind to the ATP-binding site of AC (Brand et al., 2013), selectively inhibit subtypes of AC distinct from those that mediate the effects of PGE_2_. Available antibodies do not allow quantitative assessment of the expression of AC subtypes, but QPCR analysis shows that human ASMC express similar amounts (∼30%) of AC3, AC7, AC9, some AC6 (∼10%), and detectable AC4 (∼2%) (Supplemental Fig. S6). AC9 probably does not mediate the effects of PGE_2_ on Ca^2+^ signals because AC9 is insensitive to forskolin and NKH 477 (Seifert et al., 2012), which mimic the effects of PGE_2_ on Ca^2+^ signals (Fig. 1, C–E; Fig. 2D). Among the remaining ACs expressed in ASMC, SQ22536 and DDA probably have some selectivity for AC6 over AC3 and AC7 despite some inconsistent reports (Pierre et al., 2009; Seifert et al., 2012). From analyses of individual AC isoforms, maximally effective concentrations of SQ 22356 (and other P-site inhibitors) inhibit catalytic activity by only ∼80% [Brand et al. (2013), but see Onda et al. (2001)]. This is similar to the ∼80% inhibition of PGE_2_-evoked cAMP accumulation by SQ/DDA in ASMC (Fig. 8A), suggesting that the incomplete inhibition observed in ASMC probably does not reflect the unperturbed activity of SQ/DDA-insensitive ACs. Furthermore, the effects of PGE_2_ on protein phosphorylation in ASMC are inhibited by SQ/DDA (Fig. 4B), again suggesting that the ACs activated by PGE_2_ are inhibited. We conclude that the lack of effect of SQ/DDA on PGE_2_-mediated inhibition of histamine-evoked Ca^2+^ signals is probably not the result of ineffective inhibition of an SQ/DDA-resistant subtype of AC.

To account for the results with SQ/DDA, we suggest that cAMP is delivered locally to PKA at concentrations more than sufficient to fully inhibit Ca^2+^ signals. The concentration-dependent effects of PGE_2_ might then result from recruitment of these “hyperactive” cAMP signaling junctions, rather than from increased activity within individual junctions (Fig. 8F). This interpretation is consistent with analyses of the effects of SQ/DDA on the inhibition of Ca^2+^ signals by selective activation of EP_4_ receptors. Although activation of EP_2_ and EP_4_ receptors causes similar maximal inhibition of histamine-evoked Ca^2+^ signals, EP_4_ receptors cause less stimulation of AC (Pantazaka et al., 2013). This suggests that EP_4_ receptors may less effectively saturate the cAMP signaling junctions. Whereas inhibition of AC with SQ/DDA had no effect on the inhibition of histamine-evoked Ca^2+^ signals by PGE_2_ or butaprost (to selectively activate EP_2_ receptors), the sensitivity to L902,688, a selective agonist of EP_4_ receptors, was modestly reduced by SQ/DDA (*Δ*pIC_50_ = 0.32 ± 0.10, *n* = 5) (Fig. 8, B–E). This observation supports our suggestion that the subtype(s) of AC that link prostanoid receptors to inhibition of Ca^2+^ signals are sensitive to SQ/DDA. Furthermore, these results are consistent with the scheme shown in Fig. 8F, where we suggest that cAMP is locally delivered within “hyperactive” signaling junctions at concentrations more than sufficient to maximally activate the PKA that inhibits Ca^2+^ signals.

We considered whether AKAPs, which are widely implicated in assembling PKA with its regulators and effectors (Smith et al., 2017), might contribute to organization of the cAMP signaling through PKA that leads to inhibition of histamine-evoked Ca^2+^ signals. A membrane-permeant peptide that disrupts association of AKAPs with PKA (st-Ht31) but not its inactive analog (st-Ht31P), significantly attenuated the protein phosphorylation evoked by PGE_2_, but neither peptide affected the concentration-dependent inhibition of histamine-evoked Ca^2+^ signals by PGE_2_ (Supplemental Fig. S7). These results suggest that AKAPs are probably not important components of the signaling pathway from PGE_2_ to inhibition of Ca^2+^ signals.

## Discussion

In human ASMC, the IP_3_-mediated Ca^2+^ signals evoked by activation of H_1_ histamine receptors are attenuated by PGE_2_. Several lines of evidence show that this inhibition is mediated by cAMP. The concentration-effect relationships for regulation of AC and Ca^2+^ signals by PGE_2_ are consistent with cAMP lying upstream of Ca^2+^ in the signaling pathway (Fig. 1B), direct activation of AC or membrane-permeant analogs of cAMP mimic PGE_2_, and maximal concentrations of these drugs are not additive (Figs. 1 and 2). Our conclusion that cAMP mediates the inhibition of Ca^2+^ signals in human ASMC is consistent with evidence that many receptors, via stimulation of AC, attenuate Ca^2+^ signaling in smooth muscle, including VSM (Morgado et al., 2012). Inhibition of histamine-evoked Ca^2+^ signals by PGE_2_ does not require activation of EPACs (Fig. 2, D and E). The inhibition is not mediated by accumulation of cGMP after inhibition of PDEs since neither cGMP nor inhibition of PDEs effectively mimicked PGE_2_ (Fig. 3). Inhibition of histamine-evoked Ca^2+^ signals by PGE_2_ or 8-Br-cAMP was attenuated by inhibition of PKA using H89, PKI, or *R*p-8-CPT-cAMPS (Fig. 4). We conclude that inhibition of histamine-evoked Ca^2+^ signals by PGE_2_ is (at least largely) mediated by PKA (Fig. 5D).

PKA can enhance Ca^2+^ removal from the cytosol by stimulating Ca^2+^ pumps (Tada and Toyofuku, 1998) or the Na^+^/Ca^2+^ exchanger (Karashima et al., 2007). However, accelerated removal of cytosolic Ca^2+^ does not mediate inhibition of histamine-evoked Ca^2+^ signals by PGE_2_ in human ASMC (Supplemental Fig. S5). Nor would this mechanism be consistent with the lack of effect of PGE_2_ on the Ca^2+^ signals evoked by stimulation of endogenous PAR1 or heterologously expressed M3 muscarinic receptors (Fig. 7, D–F). Cyclic AMP has been proposed to inhibit IP_3_-evoked Ca^2+^ release (Bai and Sanderson, 2006), but PKA (IP_3_R1 and IP_3_R2) and cAMP (IP_3_R1-3) more often potentiate responses to IP_3_ (Taylor, 2017). However, under conditions where PGE_2_ inhibited histamine-evoked Ca^2+^ signals, it had no effect on the sensitivity of IP_3_Rs to IP_3_ (Fig. 6). Steps linking receptors to PLC can also be inhibited by cAMP (see references in Yang et al. (1999)). Although two different assays suggested that PGE_2_ attenuated histamine-evoked PLC activity in human ASMC, neither analysis demonstrated a statistically significant effect (Fig. 7, A and B). However, the lack of effect of PGE_2_ on the Ca^2+^ signals evoked by PAR1 and muscarinic M3 receptors (Fig. 7, D–F) suggests that the inhibition of histamine-evoked Ca^2+^ signals by cAMP/PKA is probably the result of uncoupling of H_1_ histamine receptors from G_q/11_. PKA has been reported to phosphorylate H_1_ histamine receptors (Kawakami et al., 2003; Horio et al., 2004), but the functional consequences have not been thoroughly examined (Miyoshi et al., 2006). We conclude that in human ASMC, PGE_2_, through EP_2_ and EP_4_ receptors (Pantazaka et al., 2013), stimulates AC, leading to formation of cAMP and uncoupling of histamine from stimulation of PLC, most probably by PKA-mediated phosphorylation of H_1_ receptors.

Cyclic AMP can be locally delivered to intracellular targets (Zaccolo, 2011; Cooper and Tabbasum, 2014). AKAPs play prominent roles in targeting cAMP through PKA to specific cellular responses (Smith et al., 2017), but our results suggest that AKAPs probably do not contribute to inhibition of histamine-evoked Ca^2+^ signals by PGE_2_ (Supplemental Fig. S7). Our results do, however, reveal an additional complexity in the pathways linking PGE_2_ to inhibition of histamine-evoked Ca^2+^ signals. Although cAMP mediates this inhibition, the concentration-dependent effects of PGE_2_ were insensitive to substantial inhibition of AC (Fig. 8). These results and analyses of the effects of selective activation of EP_2_ and EP_4_ receptors lead to the scheme shown in Fig. 8F. We suggest that communication between EP receptors and the PKA that inhibits histamine-evoked IP_3_ formation is mediated by delivery of cAMP within signaling junctions. Activation of a junction allows local delivery of a supersaturating concentration of cAMP to PKA, allowing each junction to function as a robust on-off switch. We suggest that the concentration-dependent effects of PGE_2_ arise from recruitment of these junctions and not from graded activity within individual junctions. Such digital signaling from receptors to intracellular targets via hyperactive junctions (Fig. 8F) allows robust and reliable communication, and may be a general feature of signaling by diffusible intracellular messengers (Tovey et al., 2008).

## Abbreviations

AC: adenylyl cyclase
AKAP: A kinase-anchoring protein
ASMC: aortic smooth muscle cell
[Ca^2+^]_i_: intracellular free [Ca^2+^]
ci-IP_3_: d-2,3-*O*-isopropylidene-6-*O*-(2-nitro-4,5-dimethoxy)benzyl-*myo*-inositol 1,4,5-trisphosphate; ci-IP_3_PM, ci-IP3-hexakis(propionoxymethyl) ester
DDA: 2*′*,5*′*-dideoxyadenosine
EPAC: exchange protein activated by cAMP
ESI-09: 3-[5-(*tert*-butyl)isoxazol-3-yl]-2-[2-(3-chlorophenyl)hydrazono]-3-oxopropanenitrile
HBS: HEPES-buffered saline
HJC0197: 4-cyclopentyl-2-(2,5-dimethylbenzylsulfanyl)-6-oxo-1,6-dihydropyrimidine-5-carbonitrile
H89: *N*-[2-[[3-(4-bromophenyl)-2-propenyl]amino]ethyl]-5-isoquinolinesulfonamide dihydrochloride
IBMX: 3-isobutyl-1-methylxanthine
IP_3_: inositol 1,4,5-trisphosphate
IP_3_R: IP_3_ receptor
L902: 688 (5-[(1*E*,3*R*)-4,4-difluoro-3-hydroxy-4-phenyl-1-buten-1-yl]-1-[6-(2*H*-tetrazol-5*R*-yl)hexyl]-2-pyrrolidinone)
NKH 477: (*N*,*N*-dimethyl-(3*R*,4a*R*,5*S*,6a*S*,10*S*,10a*R*,10b*S*)-5-(acetyloxy)-3-ethenyldodecahydro-10,10b-dihydroxy-3,4a,7,7,10a-pentamethyl-1-oxo-1*H*-naphtho[2,1-*b*]pyran-6-yl ester *β*-alanine hydrochloride)
PAR1: protease-activated receptor type 1
PDE: cyclic nucleotide phosphodiesterase
pIC_50_ (pEC_50_): negative logarithm of the half-maximally inhibitory (effective) concentration
PGE_2_: prostaglandin E_2_
PKA: cAMP-dependent protein kinase
PKG: cGMP-dependent protein kinase
PKI (mut PKI): PKA inhibitor peptide (mutant inactive form)
PLC: phospholipase C
SQ 22536: 9-(tetrahydro-2-furanyl)-9*H*-purin-6-amine
SQ/DDA: 1 mM SQ 22536 with 200 *μ*M DDA
TBS: Tris-buffered saline
TBS-T: TBS with Tween
VASP: vasodilator-stimulated phosphoprotein
VSM: vascular smooth muscle
